# A Case of Primary Intramedullary Infantile Fibrosarcoma With an *RBPMS::NTRK3* Fusion and Response to Larotrectinib

**DOI:** 10.1111/nan.70102

**Published:** 2026-10-04

**Authors:** Mégane Le Quang, Julie Tandonnet, Nathalène Truffaux, Lucile Vanhersecke, Jean Delmas, Arthur Durouchoux, Dorian Bochaton, Arnault Tauziède‐Espariat, Guillaume Chotard

**Affiliations:** ^1^ Department of Pathology University Hospital of Bordeaux Bordeaux France; ^2^ Pediatric Hematology and Oncology Unit University Hospital of Bordeaux Bordeaux France; ^3^ Institut Bergonié, Department of Biopathology Bordeaux France; ^4^ Department of Radiology University Hospital of Bordeaux Bordeaux France; ^5^ Department of Neurosurgery University Hospital of Bordeaux Bordeaux France; ^6^ Department of Neuropathology, GHU Paris ‐ Psychiatry and Neuroscience Sainte‐Anne Hospital Paris France; ^7^ INSERM U1339/CNRS UMR 3666, Chemical Biology of Cancer, Institut Curie Paris France

**Keywords:** central nervous system tumour, infantile fibrosarcoma, intramedullary tumour, larotrectinib, *RBPMS::NTRK3* fusion

## Abstract

- First reported case of primary central nervous system infantile fibrosarcoma.
- Expansion of the molecular spectrum of infantile fibrosarcoma with identification of an *RBPMS::NTRK3* fusion.
- Marked efficacy of larotrectinib observed in an unresectable CNS tumour.
- Reinforces the importance of molecular testing in atypical paediatric CNS tumours.

## Graphical abstract

We report a case of primary intramedullary infantile fibrosarcoma (IFS) in a 4‐week‐old infant with rapidly progressive cervical myelopathy. Histology showed a spindle cell tumour with diffuse S100 and pan‐TRK expression. RNA sequencing identified an *RBPMS::NTRK3* fusion. RNA‐based clustering and DNA methylation profiling classified the tumour as IFS. Treatment with the TRK inhibitor, larotrectinib, achieved marked clinical and radiological recovery within 1 month, expanding the spectrum of *NTRK*‐rearranged sarcomas and confirming TRK inhibition's efficacy in primary central nervous system IFS.

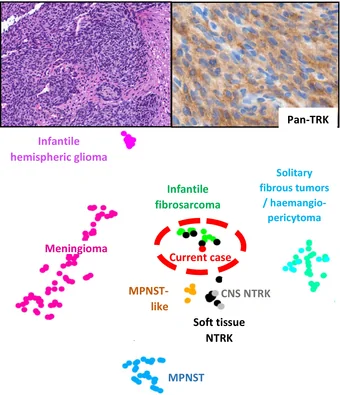

## 1 Case Report

Infantile fibrosarcoma (IFS), the most common soft tissue sarcoma of early childhood, is typically driven by an *ETV6*::*NTRK3* fusion and arises in the extremities, with prognosis largely determined by its locally aggressive behaviour [1]. Management relies on conservative surgery, with chemotherapy (most commonly vincristine and dactinomycin) used in unresectable cases [2]. Although TRK inhibitors such as larotrectinib have shown high efficacy in *NTRK*‐rearranged tumours, they are not yet standard first‐line therapy for IFS [3, 4]. Here, we report the first documented case of primary central nervous system (CNS) IFS, with a rapid clinical and radiological response to larotrectinib within 1 month.

A 4‐week‐old infant was admitted to the paediatric emergency department with rapidly progressive flaccid weakness of the upper limbs. The child was born at 38 weeks of gestation after induction for intrauterine growth restriction. The neonatal examination had been unremarkable except for right‐sided congenital torticollis. There was no family history of neurological disorders. MRI of the brain and spinal cord revealed an expansile, infiltrative intramedullary lesion extending from C3 to T2 over approximately 35 mm in height and 15 mm in anteroposterior diameter. The mass was isointense on T2‐weighted imaging, slightly hypointense on T1‐weighted sequences and showed homogeneous contrast enhancement. Comprehensive imaging, including brain and spine MRI and thoraco‐abdomino‐pelvic CT, revealed no other tumour lesions, supporting a primary intramedullary tumour. Given the rapid neurological decline, corticosteroid therapy was initiated, without clinical improvement. Because surgical excision was not feasible, a large open biopsy was performed.

Histopathological examination revealed a tumour with morphology reminiscent of IFS, characterised by a densely cellular proliferation arranged in intersecting fascicles of monotonous spindle cells within a sparsely collagenous stroma (Figure 1A). The neoplasm diffusely infiltrated the surrounding spinal cord parenchyma, entrapping residual neural tissue (Figure 1B). The tumour cells displayed elongated nuclei with fine chromatin and indistinct eosinophilic cytoplasm (Figure 1C). The vascular network consisted of thin‐walled, ectatic vessels. No necrosis or inflammatory infiltrate was identified. The mitotic index reached eight mitoses per mm^2^. Immunohistochemistry showed diffuse S100 positivity (Figure 1D) and diffuse intracytoplasmic pan‐tropomyosin receptor kinase (pan‐TRK) staining (Figure 1E,F), whereas anti‐TRK‐C was negative. Alpha‐smooth muscle actin and CD34 showed heterogeneous expression. Markers of glial (GFAP and OLIG2), neuronal (neurofilament and SOX10), epithelial (cytokeratin AE1/AE3 and EMA) and muscular differentiation (desmin, caldesmon and myogenin) were negative. Trimethylation of H3K27 was retained. The Ki‐67 labelling index reached 40%.

**FIGURE 1:**
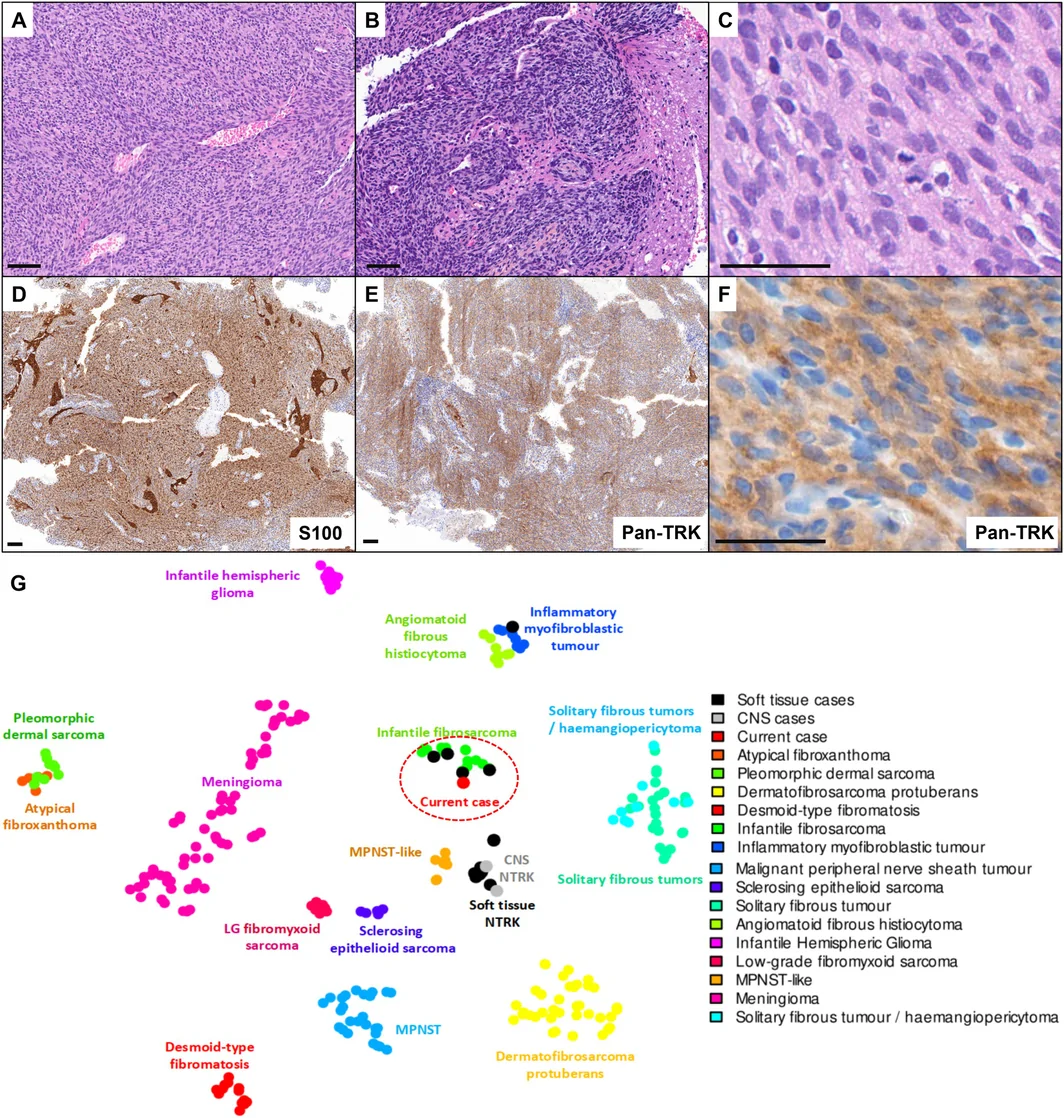
Histopathological and immunohistochemical features with methylation‐based t‐SNE distribution. Histological examination revealed a densely cellular spindle cell neoplasm arranged in intersecting fascicles (A), with entrapment of residual neural tissue (B). The tumour cells were monotonous, with elongated nuclei and fine chromatin (C). Immunohistochemistry demonstrated diffuse S100 positivity (D) and diffuse intracytoplasmic pan‐TRK staining (E‐F). Scale bars: 100 μm. T‐distributed stochastic neighbour embedding (t‐SNE) analysis of DNA methylation profiles included reference samples from brain and soft tissue tumours: 5 angiomatoid fibrous histiocytomas, 5 atypical fibroxanthomas, 36 dermatofibrosarcomas protuberans, 13 desmoid‐type fibromatoses, 14 infantile fibrosarcomas (IFS), 9 infantile hemispheric gliomas, 7 inflammatory myofibroblastic tumours, 8 low‐grade fibromyxoid sarcomas, 25 malignant peripheral nerve sheath tumours, 67 meningiomas, 7 malignant peripheral nerve sheath tumours (MPNST)‐like, 9 pleomorphic dermal sarcomas, 6 sclerosing epithelioid sarcomas, 26 solitary fibrous tumours, 9 solitary fibrous tumours/haemangiopericytomas and 16 *NTRK*‐rearranged spindle cell neoplasms, including 14 arising in soft tissues and 2 in the brain. Our case segregates with the IFS group (G).

RNA sequencing was performed using the Illumina TruSeq RNA Exome kit on a NextSeq 500 platform, with 98.3% theoretical RefSeq exome coverage. The *RBPMS*::*NTRK3* fusion was consistently identified by four fusion‐calling tools: STAR‐Fusion, FusionMap, Arriba and FusionCatcher, supported by 348, 49, 236 and 137 junction reads, respectively. *NTRK3* expression was 39.90 reads per kilobase million (RPKM).

Whole‐genome and whole‐exome sequencing were performed by the AURAGEN platform within the France Médecine Génomique (FMG) programme and revealed no other molecular alterations. Unsupervised hierarchical clustering of RNA‐sequencing expression profiles placed the tumour within a cluster comprising IFS, *NTRK*‐rearranged fibroblastic tumours and uterine *NTRK*‐rearranged sarcomas. DNA methylation‐based classification using brain tumour classifiers assigned the tumour to the mesenchymal tumour group, within the atypical malignant peripheral nerve sheath tumour (MPNST) subclass, with calibrated scores of 0.99 and 0.70 for the main class in the Bethesda Brain Tumour Classifier v2.0 and the Epignostix CNS Tumour Methylation Classifier v12.8, respectively. In contrast, the Epignostix Sarcoma Classifier v13.1 classified the lesion as IFS, with a score of 0.40. T‐distributed stochastic neighbour embedding (t‐SNE) analysis of DNA methylation profiles from our case and selected reference brain and soft tissue tumours, including MPNST, IFS and *NTRK*‐rearranged tumours, was performed to visualise similarities among these entities. Our case segregated with the IFS group (Figure 1G).

Although molecular results were awaited, the infant had a clinical and radiological deterioration, with the onset of quadriplegia. Therefore, vincristine, actinomycin and cyclophosphamide (VAC) chemotherapy was initiated 2 weeks after the biopsy. MRI performed 1 month after biopsy demonstrated progression of the lesion, extending from C2 to the inferior endplate of T3 over approximately 45 mm, with effacement of the perimedullary cerebrospinal fluid rim (Figures 2A,B). Following molecular confirmation of an *NTRK* fusion 1 month after biopsy, and in the absence of clinical improvement after 2 weeks of VAC therapy, treatment was switched to larotrectinib at 50 mg/m^2^ twice daily until 3 months of age and then increased to 100 mg/m^2^ twice daily. After 15 days of treatment, ventilatory support through the tracheostomy was successfully weaned. Within 1 month, the infant regained spontaneous lower‐limb movements and showed partial improvement in upper‐limb motor function, with residual distal spasticity. Decannulation was performed after 2 months of treatment. Follow‐up MRI demonstrated a marked reduction of the intramedullary lesion, with reappearance of perimedullary cerebrospinal fluid spaces (Figure 2C). Only faint residual T1 hypointensity persisted, with minimal contrast enhancement. The last follow‐up, 6 months after treatment initiation, demonstrated a sustained favourable radiological response, with almost complete restoration of normal spinal cord calibre (Figure 2D). The clinical course was similarly favourable, with ongoing catch‐up in developmental milestones. No treatment‐related adverse events were observed. Therapy will continue for at least 12 months, after which continuation will be reassessed based on clinical response and tolerability.

**FIGURE 2:**
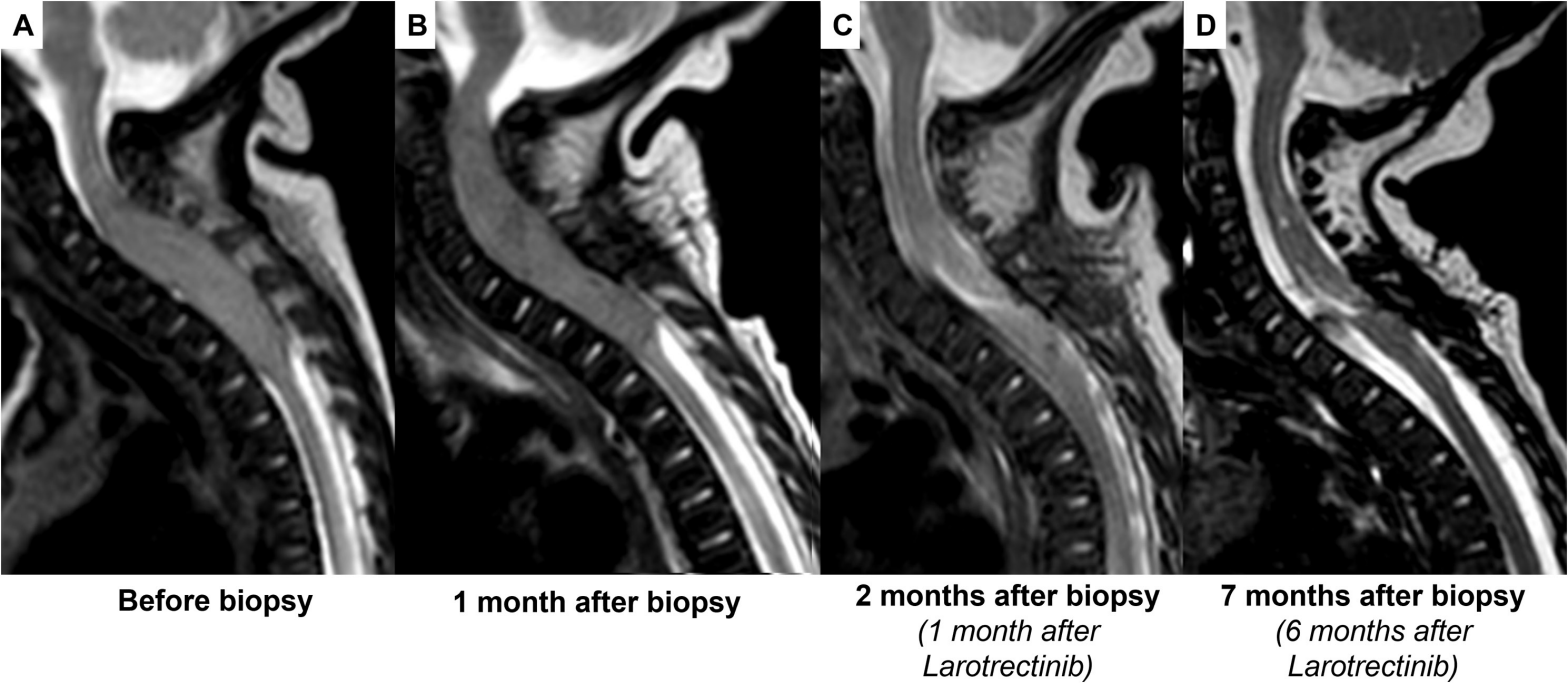
MRI characterisation and evolution of the tumour. T2‐weighted MRI sequence showing an isointense, infiltrative intramedullary lesion extending from C3 to T2 (approximately 35 mm) (A). One month after the biopsy, the lesion extended from C2 to the inferior endplate of T3 (approximately 45 mm), with effacement of the perimedullary cerebrospinal fluid rim (B). One month after the introduction of larotrectinib (2 months after biopsy), follow‐up MRI demonstrated marked reduction of the lesion and reappearance of perimedullary cerebrospinal fluid spaces (C). At 6 months after treatment initiation (7 months after biopsy), MRI showed a sustained favourable response, with almost complete restoration of normal spinal cord calibre (D).

Although rare metastatic CNS locations have been described, no primary CNS IFS has been reported, to our knowledge. Comprehensive CNS and systemic imaging identified no additional lesions, supporting this as the first reported primary CNS IFS.

Despite the spindle cell morphology, the isolated intramedullary location initially favoured a primary CNS tumour, particularly a paediatric glial tumour or infantile myofibromatosis. These diagnoses were investigated first, but the tumour was negative for glial and muscular markers. Although high‐grade, particularly sarcomatoid, glial tumours may lose glial marker expression, IFS was considered based on the morphological resemblance and pan‐TRK immunohistochemistry was performed, revealing strong expression and prompting molecular confirmation of an *NTRK* fusion.

Although rare *NTRK*‐rearranged spindle cell tumours have previously been described in the CNS [5], DNA methylation profiling distinguished our tumour from this entity and demonstrated clustering with conventional soft tissue IFS. Moreover, our case lacked the homozygous *CDKN2A* deletion typically observed in CNS *NTRK*‐rearranged spindle cell tumours [5].

The cellular origin of IFS remains unclear, with perivascular mesenchymal stem cells and sparse stromal populations, including meningeal and perivascular fibroblasts, representing plausible sources within the CNS [6].

The *RBPMS*::*NTRK3* fusion identified in our patient has not been described in IFS but has been reported in other mesenchymal neoplasms with *NTRK* rearrangements [7], including a CNS tumour lacking the morphological features of classic IFS [8] and uterine fibrosarcomas [9]. Its oncogenicity is supported by high fusion‐transcript expression, the absence of other pathogenic alterations on comprehensive RNA, whole‐genome and whole‐exome sequencing and the major clinical and radiological response to TRK inhibition.

RBPMS (RNA‐binding protein with multiple splicing) is an RNA‐binding protein of the RRM (RNA recognition motif) family, whereas *NTRK3* encodes a receptor tyrosine kinase involved in neural development. Preservation of *NTRK3* exons 15–20 is sufficient for downstream TRK signalling, and most of the RBPMS RNA‐binding domain is encoded by exons 2–5. The *RBPMS* (exon 5)::*NTRK3* (exon 12) fusion is therefore predicted to constitutively activate the *NTRK3* kinase domain and the PI3K–AKT pathway, promoting tumour cell proliferation and survival [8].

Our patient underwent partial surgical resection, followed by progression under VAC chemotherapy, prompting an early switch to larotrectinib after molecular confirmation of an *NTRK* fusion. Larotrectinib was selected because of its paediatric approval and availability as an oral suspension, making it particularly suitable for young children. Within 1 month, a marked clinical and radiological improvement was observed, consistent with the reported high efficacy of TRK inhibition in *NTRK*‐fusion tumours [3, 4]. Larotrectinib will be continued for at least 12 months before re‐evaluation, in accordance with Food and Drug Administration (FDA) recommendations to continue treatment until disease progression or unacceptable toxicity. However, longer‐term discontinuation may be considered after a sustained response, although approximately one‐third of patients subsequently relapse. Most regain disease control upon larotrectinib rechallenge [10]. TRK inhibitors have demonstrated clinically meaningful intracranial activity, including in paediatric gliomas harbouring *NTRK* alterations, supporting their ability to achieve therapeutically relevant concentrations within the CNS [11]. By contrast, vincristine exhibits poor CNS penetration in humans [12] and rat models [13]. Dactinomycin similarly shows minimal diffusion across the blood–brain barrier [14].

In summary, this case broadens the recognised anatomic spectrum of IFS to include primary CNS lesions and extends its molecular landscape to *RBPMS::NTRK3* fusions. This observation is of diagnostic relevance, as the morphology may be non‐specific and overlap with several differential diagnoses, including sarcomatoid paediatric diffuse gliomas, embryonal tumours and myofibromatosis. Although immunophenotyping may help distinguish these entities, CNS biopsy material is often limited, constraining the immunohistochemical and molecular work‐up. In this context, pan‐TRK immunostaining may be particularly informative: Although previously shown to lack specificity in brain tumours [15], strong and diffuse staining should prompt investigation for an *NTRK* fusion.

Finally, this case underscores the potential value of prioritising larotrectinib as an early therapeutic option for unresectable CNS‐localised IFS, given the limited CNS penetration of VA chemotherapy, which is the standard first‐line therapy for unresectable non‐CNS IFS [2].

## Author Contributions

M.L.Q. and G.C. drafted the manuscript. J.T. was responsible for the clinical care of the patient. M.L.Q., G.C. and L.V. conducted the histopathological analysis. N.T., D.B. and A.T.‐E. conducted the molecular analyses. a.d. performed the neurosurgical procedure. J.D. conducted the radiological analysis. All authors critically reviewed the manuscript, approved the final version and agreed to be accountable for all aspects of the work.

## Funding

The authors have nothing to report.

## Ethics Statement

Written informed consent was signed by the legal guardian for publication.

## Consent

Written informed consent was obtained from the patient's legal guardians for the use of clinical, radiological and molecular data for research and publication.

## Conflicts of Interest

The authors declare no conflicts of interest.

## Data Availability

The data that support the findings of this study are available on request from the corresponding author. The data are not publicly available due to privacy or ethical restrictions.
